# Rifaximin-induced changes in the gut microbiome associated to improvement of neurotransmission alterations and learning in rats with chronic liver disease

**DOI:** 10.1038/s41598-025-17229-1

**Published:** 2025-10-02

**Authors:** Lola Giner-Pérez, Víctor Hugo Jarquín-Díaz, Paola Leone, Carla Giménez-Garzó, Gergana Mincheva, Álex Mira, Sofia Kirke Forslund-Startceva, Teresa Rubio, Vicente Felipo, Gaspar Pérez-Martínez, Marta Llansola

**Affiliations:** 1 Lactic acid bacteria and probiotics Laboratory, Instituto de Agroquímica y Tecnología de Alimentos, Spanish National Research Council (CSIC), Paterna, Valencia, Spain; 2 Laboratory of Neurobiology, Centro de Investigación Príncipe Felipe (CIPF), Valencia, Spain; 3 Max-Delbrück-Center for Molecular Medicine in the Helmholtz Association (MDC), Berlin, Germany; 4 Charité – Universitätsmedizin Berlin, corporate member of Freie Universität Berlin and Humboldt-Universität zu Berlin, Experimental and Clinical Research Center, Lindenberger Weg 80, 13125 Berlin, Germany; 5 Experimental and Clinical Research Center, a cooperation between the Max-Delbrück-Center for Molecular Medicine in the Helmholtz Association and the Charité - Universitätsmedizin Berlin, Berlin, Germany; 6 Genomics and Health Department, FISABIO Foundation, Valencia, Spain; 7 Present Address: Icahn School of Medicine at Mount Sinai, New York, USA; 8 Present Address: Hospital de la Rivera, Alcira, Valencia, Spain

**Keywords:** Hepatic encephalopathy, Microbiome diversity, Butyric acid, Host-microbe interaction, Rifaximin, Computational biology and bioinformatics, Microbiology, Systems biology, Biomarkers, Diseases, Gastroenterology, Neurology

## Abstract

Rifaximin, a gut-targeted antibiotic, improves cognitive function and reduces the risk of hepatic encephalopathy (HE), yet its effects on the gut-brain axis remain unknown. This study explores how rifaximin influences gut microbiota functions and its association with cognitive function and molecular alterations in rats with liver injury. Liver injury was induced by chronic administration of carbon tetrachloride (CCl4), and rifaximin was administered daily. Fecal samples were collected after eight weeks of CCl4 administration, and taxonomic and functional changes in the gut microbiome were analyzed. Rifaximin altered microbiota diversity and composition, increasing α diversity in liver-injured rats but reducing diversity in healthy rats. It influenced microbiota interactions with neurotransmission alterations, where *Dorea*, *Lachnospiraceae A2*, and possibly *Erysipelotricaceae* might be important contributors. Functionally, butyric acid levels negatively correlated with gene orthologues associated with GABA, tryptophan, and glutamate degradation pathways. In healthy rats, fecal short-chain fatty acid (SCFA) levels were positively correlated with each other, a pattern absent in other groups. Rifaximin significantly influenced gut microbiota and promoted bacterial groups linked to improved cognition and neurotransmission in liver disease. Our findings underscored the direct relationship between a healthy microbiome and the maintenance of balanced SCFA concentrations.

**Supplementary Information:**

The online version contains supplementary material available at 10.1038/s41598-025-17229-1.

## Introduction

In recent years, research has highlighted that neurological impairment associated with liver disease is profoundly influenced by the gut microbiome^1^. Hepatic encephalopathy (HE) exemplifies how the gut-liver-brain axis plays a pivotal role in linking gut health with brain function in liver disease. HE is a complex neuropsychiatric syndrome that affects patients with liver cirrhosis and can range from mild symptoms to life-threatening coma and death. HE affects a significant proportion of cirrhotic patients, posing major health, social and economic challenges. Patients without evident HE symptoms may present minimal HE (MHE). These less-evident symptoms can be revealed using psychometric tests. Cirrhotic patients with MHE show high incidence of mild cognitive impairment, attention deficits and psychomotor slowing and this can progress to clinical HE^2^.

Chronic administration of the hepatotoxin carbon tetrachloride (CCl_4_) is a usual model of progressive liver injury in rats^3–6^. Previous studies using this model reported motor incoordination and cognitive impairment reproducing symptoms of MHE patients, already in a stage of mild liver injury, similar to that of patients with steatotic liver disease (SLD), including patients with no alcoholic fatty liver disease or steatohepatitis. These neurological impairments in CCl_4_-induced mild liver injury were associated with peripheral inflammation, promoting immune cells infiltration in the cerebellum and hippocampus, leading to neuroinflammation and altered neurotransmission in these cerebral areas^5,6^.

Treatment with rifaximin, a non-systemic antibiotic that primarily targets the gut, is effective in restoring cognitive function in cirrhotic patients with MHE and reduces the risk of recurrent HE. This was associated with a shift in peripheral inflammation^7,8^, suggesting HE symptoms may lie downstream of gut microbiome alterations in cirrhosis. Rifaximin treatment in CCl_4_ treated rats reduced peripheral inflammation and neuroinflammation, improving motor and cognitive function^5,6^. These aforementioned studies demonstrated that rifaximin treatment reduced extracellular GABA levels in the cerebellum by modulating membrane expression of GABA transporters, which mediated improving of motor coordination in rats with mild liver damage induced by CCl_4_ administration. These effects of rifaximin are mediated mainly by reduction of the increased chemokines in peripheral blood, and in brain, which reduced immune cell infiltration and subsequent neuroinflammation, including TNFα proinflammatory cytokine increase in the cerebellum^5^. In the same way, the administration of rifaximin also reduced hippocampal neuroinflammation, which normalized membrane expression of the glutamate NMDA receptor subunits. This, in turn, contributed to improvement of cognition, specifically spatial learning and memory (novel object location memory and learning in the radial maze)^6^. The precise mechanisms underlying these beneficial effects of rifaximin on inflammation and brain function in rats with mild liver damage remain to be elucidated, but modulation of the gut microbiome may be involved. This modulation may occur through metabolic interactions^9^ or by promoting a gut microenvironment with cytoprotective properties^10^. Nevertheless, the effect of rifaximin on the human microbiome is a major goal of ongoing research, but until present, results are still contradictory. Some studies on human samples have shown that this antibiotic minimally affects the composition of the microbiota of HE patients^11,12^, while other work^13^ found a significant decrease in the α-diversity of the fecal microbiome, as well as in oral microbiota, but without finding significant changes in composition (β-diversity).

The role of the gut microbiome can be studied using different approaches, either by analyzing microbial taxonomy or by examining the metabolic capacity and activity of the microbes. The taxonomic composition provides valuable information on the microbiome status, as certain groups are often associated with health or disease status. Microbiota taxonomic groups are associated with several groups of microbiota-derived metabolites that are responsible for most microbiota effects on host physiology. Metabolites like short-chain fatty acids (SCFA) have a key role mediating the microbiota-host interaction. SCFA are produced as major fermentation products of certain bacterial groups from dietary fiber in the gut. The production of SCFA is a highly relevant functional aspect of the microbiome, as they are considered key factors in gut-brain axis communication^14,15^. Yet, few studies have investigated the role of SCFA as potential mediators for microbiome-focused interventions that influence cognitive functioning^16^.

The selective effect of rifaximin on the gut microbiome is essential to understand the intricate relationship between the gut microbiome and liver disease, which is required to develop new approaches for the treatment of MHE. In this work, the effect of rifaximin on the gut microbiome was evaluated in the rat model of CCl_4_-induced liver injury to determine specific changes in the composition of the gut microbial populations and their functionality. In addition, potential relationships were identified between SCFA and disease-related metabolic pathways.

## Results

### Rifaximin is the primary factor influencing microbial diversity in the rodent MHE model

**Fig. 1:**
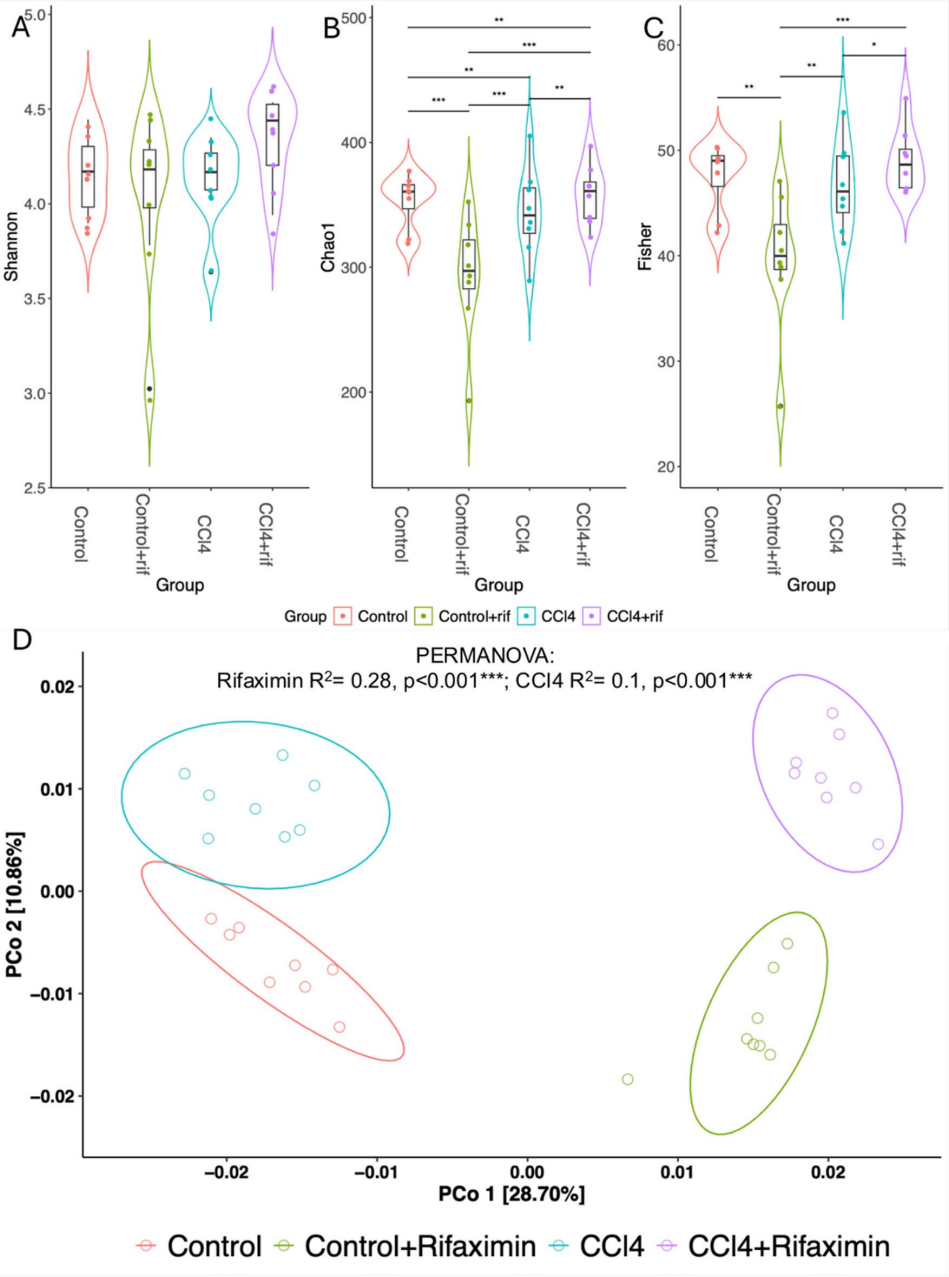
Impact of rifaximin treatment on gut microbiome diversity in healthy and mild liver-injured rats. Box plots illustrating α-diversity indexes Shannon diversity (A), Chaotropic 1 index (B), and Fisher index (C) in bacterial microbiomes of fecal samples from healthy rats (Control), healthy rats treated with rifaximin (Control + rif), rats with induced liver injury (CCl_4_), rats with induced liver injury treated with rifaximin (CCl_4_ + rif) (*n* = 8 per group). Adjusted p-values of likelihood ratio test of nested models: <0.001 = ***, < 0.01 = **, < 0.05 = *. Principal Coordinates Analysis (PCoA) of Bray-Curtis distances (D). Distances were used to compare the taxonomic and functional diversity of gut microbiomes between populations. The axis labels show the proportion of variance explained by each principal coordinate axis. PERMANOVA for the group variable significance and R2 for antibiotic and CCl_4_ treatment variables are indicated on the top area of the plot.

To study the changes induced by rifaximin on the gut microbiota, fecal samples of four experimental rat groups (*n* = 8 per group) were collected at a single timepoint. These experimental groups were two control groups, with and without rifaximin, and two groups with induced liver injury, with and without rifaximin treatment. Gut microbiota diversity, evenness, and richness were estimated as alpha α-diversity parameters for the microbial communities of the experimental groups (Fig. 1, Supplementary Fig. 1). Statistical comparisons between the groups were performed to assess differences, accounting for batch-related effects using linear mixed models. No significant differences in diversity (Shannon index) were found between the groups (Fig. 1A) (Likelihood Ratio Test -LRT-, *Χ*^2^ = 1.31, *p* = 0.73). However, significant differences in richness when applying Chao1 α-index (Fig. 1B) and in evenness when applying Fisher’s α-index (Fig. 1C) between groups were observed (LRT, Fisher: *Χ*^2^ = 26.964, *p* < 0.001; Chao1: *Χ*^2^ = 37.343, *p* < 0.001). Interestingly, a reduction in diversity was observed after rifaximin treatment in healthy rats, whereas in rats with mild liver injury rifaximin moderately increased in both α-diversity indices.

Then, Principal Coordinates Analysis (PCoA) ordination of Bray-Curtis distance matrices was used for the visualization of the microbial community structure (β-diversity) (Fig. 1D). A clear separation, mainly explained by PCoA1, was observed under treatment with rifaximin, indicating that antibiotic treatment is the main factor affecting microbiota diversity in this model (PERMANOVA, R2 = 0.27840, *p* < 0.001). There were also significant differences between healthy rats and rats with liver injury (PERMANOVA, R2 = 0.10037, *p* < 0.001), indicating that the microbiome was compositionally different in these groups. The reliability of our results was ensured by verifying that there were no statistical differences due to homogeneity of variance between the groups (ANOVA, F value = 0.4711, *p* > 0.05).

### Rifaximin treatment induced more taxonomical changes in to rats with liver injury (CCl_4_) compared to healthy rats

**Fig. 2:**
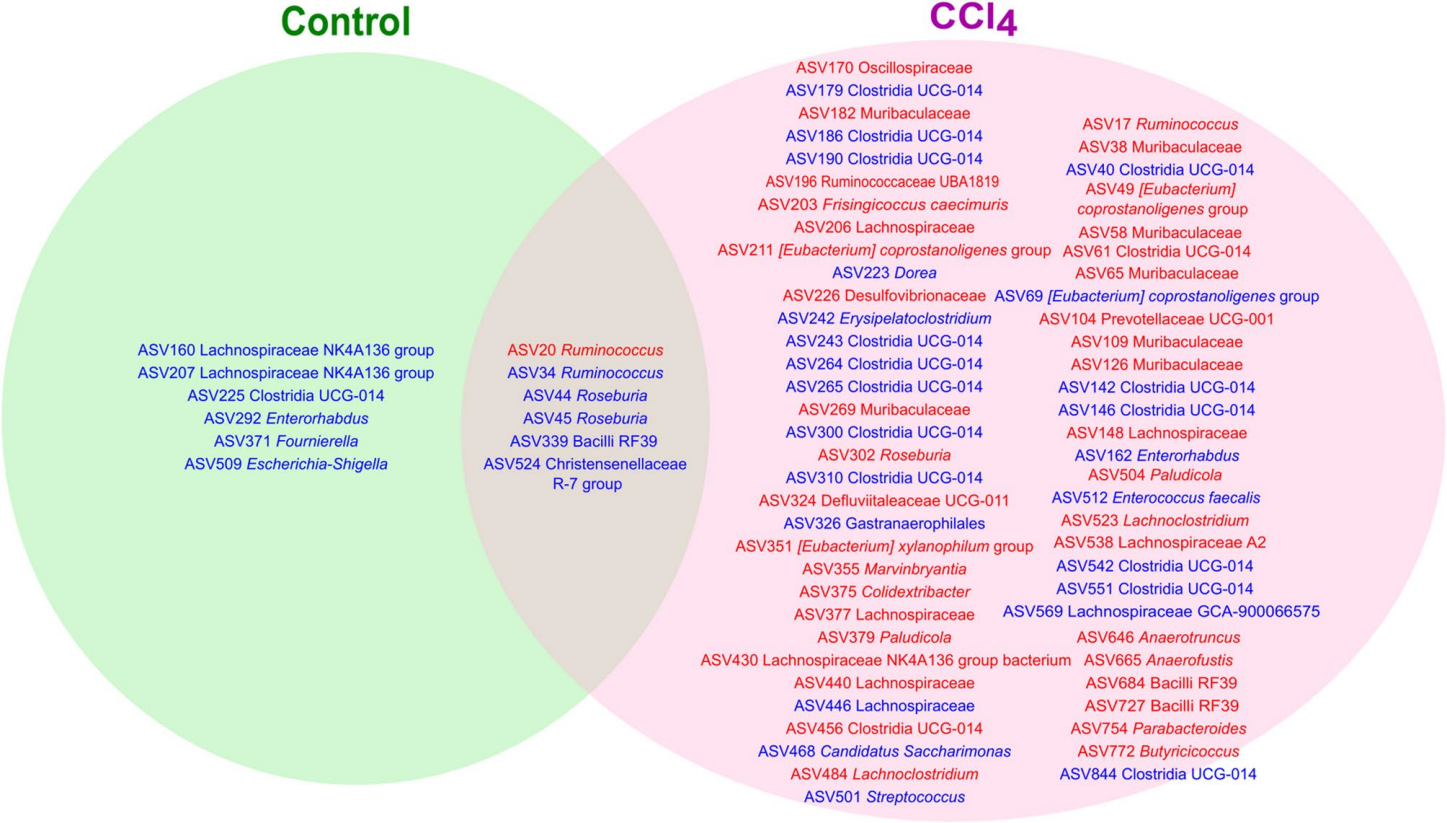
Venn Diagram displaying ASVs associated with rifaximin treatment by group. This figure displays the significant relationships between ASVs and rifaximin treatment in healthy rats and rats with mild liver injury (CCl4), tested separately (*n* = 8 per group). The colors represent if the correlation between the ASV and rifaximin was positive (red) or negative (blue).

The number of bacterial amplicon sequence variants (ASVs) positively or negatively associated with rifaximin treatment differed between healthy rats and rats with liver injury. The associations described in this section refer specifically to statistically significant (p-value < 0.01) associations between rifaximin treatment and the direction of change in microbial compositional abundance of each ASV, assessed using metadeconfoundR for each group independently. In healthy rats, 12 ASVs showed associations with rifaximin treatment, whereas 68 ASVs were associated with the antibiotic treatment in rats with mild liver injury. In healthy rats, rifaximin was associated with reduction of ASVs belonging to the families *Lachnospiraceae*, *Ruminococcaceae*, *Eggerthellaceae*, *Christensenellaceae*, *Enterobacteriaceae*, one ASV from group Clostridia UCG-014 and other from *Bacilli* group RF39. Only one positive association was found in healthy rats between rifaximin and an ASV belonging to the family *Ruminococcaceae*. In rats with liver injury, the antibiotic treatment had positive and negative associations with 68 ASVs, showing a more complex effect on the changes of bacterial groups (Fig. 2, Supplementary Fig. 2, Supplementary Files 2 and 3). As indicated above in healthy rats, almost all taxonomical associations with rifaximin observed were negative, indicating a decrease in microbial abundance in response to the antibiotic, which is also supported by the results in α-diversity.

### Rifaximin is the primary confounding factor when assessing taxonomic changes correlated with Neurobiological and metabolic measures

Immunohistochemistry data, receptors and cytokines expression in the hippocampus and learning and memory tests data were obtained from rats used in our previously published work^5,6^ and additional method information is described in the Supplementary methods file. Metadata can be found in the Supplementary file 1.

**Fig. 3:**
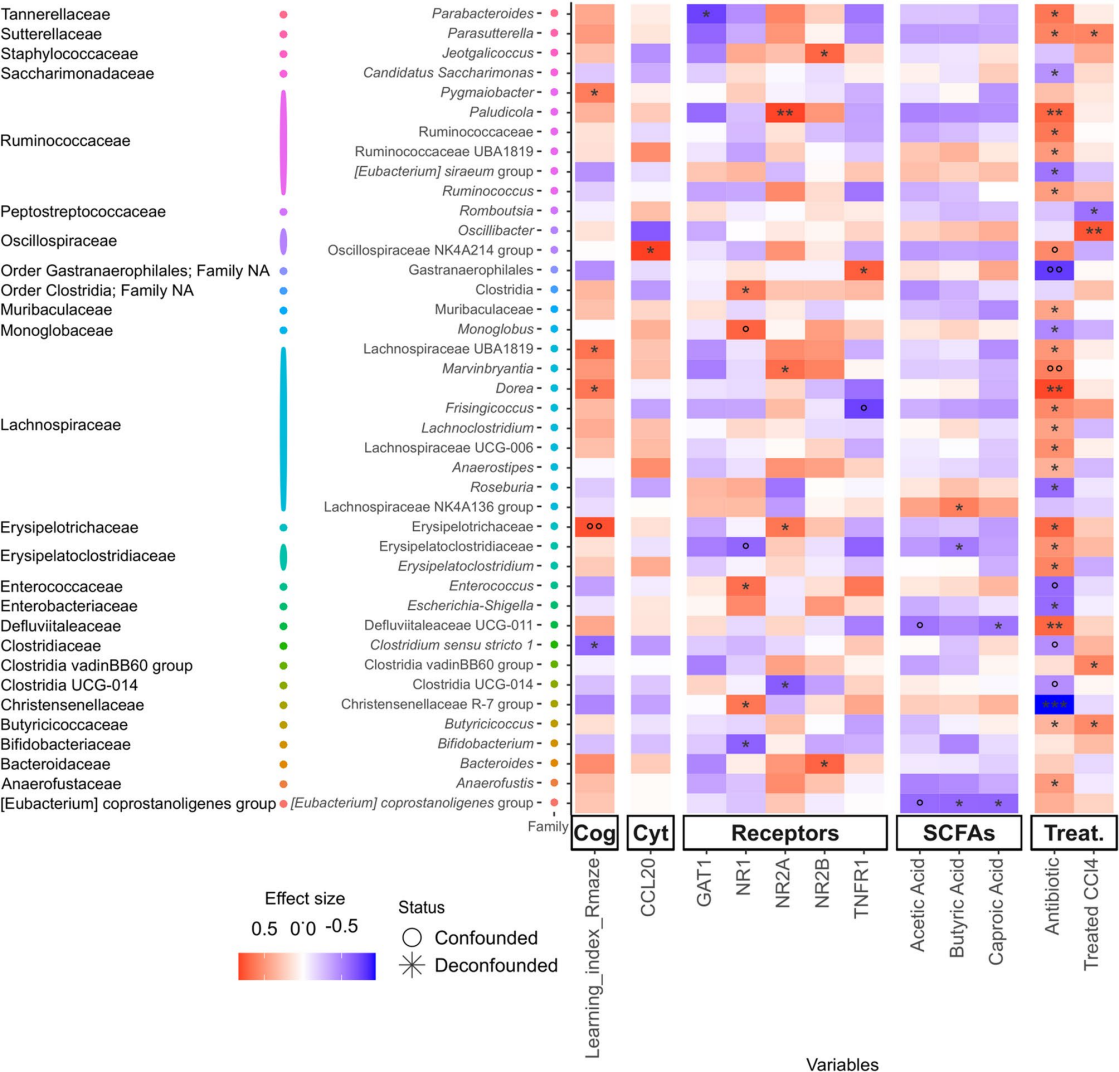
Heatmap showing the significant association between bacterial genera and variables. Significant relationships between ASVs agglomerated by genus and metadata variables, on the y and x-axis respectively. The color scale on the heatmap represents the magnitude of the effect size, and the color scale on the y-axis represents the Families each taxon belongs to. The significance of each association is represented by black asterisks based on the FDR-adjusted p-values of the initial tests (FDR-values: < 0.001 = ***, < 0.01 = **, < 0.1 = *). Associations that are significant but confounded are represented by gray circles instead. SCFA, short chain fatty acids; Cog, cognition; Cyt, cytokines, Treat, treatment.

Significant correlations between microbial genera abundance and metadata variables were identified, while also assessing which of these associations were due to a potential confounding effect (Fig. 3, Supplementary Fig. 3). Rifaximin treatment had the strongest effect on microbiota composition and abundance. This strong effect of rifaximin on bacterial genera had a projection on the association of microbial genera with other variables. Using the metadeconfoundR tool for this analysis allowed us to find associations that could be influenced by potential confounders. It is important to statistically determine when the association between two variables, for example the bacterial genus A and membrane receptor X or learning index, is confounded by a third factor (e.g., rifaximin), because this shows that rifaximin has a strong influence on the bacterial genus A. In this case it is important to show the selective effect of this antibiotic and it explains the possible mechanism by which rifaximin indirectly is affecting the expression of brain receptors and cognitive faculties (Supplementary Table 4). For instance, in the presence of rifaximin, *Erysipelotrichaceae* positive association to cognition (learning index assessed in the radial maze test) is confounded by its (positive) association to receptor NR2A, underscoring the known connection of the expression of NR2A with cognition, and which may relate *Erysipelotricaceae* with improved cognition in the presence of rifaximin (Supplementary Table 4). *Erysipelotricaceae* is a family of intestinal commensal anaerobic bacteria belonging to the *Firmicutes* phylum. This would also explain the positive non-confounded associations between other bacterial genera, such as *Lachnospiraceae A2* and *Dorea*, with cognition in the presence of rifaximin (Fig. 3). *Dorea* as well as other *Firmicutes* bacteria in the *Lachnospiraceae* family are gut bacteria highly involved in the production of SCFA. It must be underlined that significant association does not necessarily relate to causality. Finally, the associations between acetic acid and various genera are confounded by other SCFA.

The levels of 16 cytokines in the hippocampus were measured to assess neuroinflammatory signaling; however, only CCL20 levels showed a significant association with the genus *Lachnospiraceae* NK4A136 group (Fig. 3). Significant associations between genera and membrane protein expression profiles were observed (Fig. 3). Looking at the general association patterns of bacterial genera, it is evident that the column showing the antibiotic pattern has the highest effect sizes and is very similar to the cognition column and to the expression patterns of NR2A and NR2B membrane receptor subunits, while the TNFα membrane receptor, TNFR1, showed a complementary or opposite association pattern. Other neurotransmitters also showed the pattern observed for TNFR1, such as the NMDA receptor subunit NR1 and the GABA transporter GAT1, opposite to the pattern observed with antibiotic treatment.

### Predicted functional metabolic modules in the microbiota showed strong correlations with fecal Butyric acid concentration

**Fig. 4:**
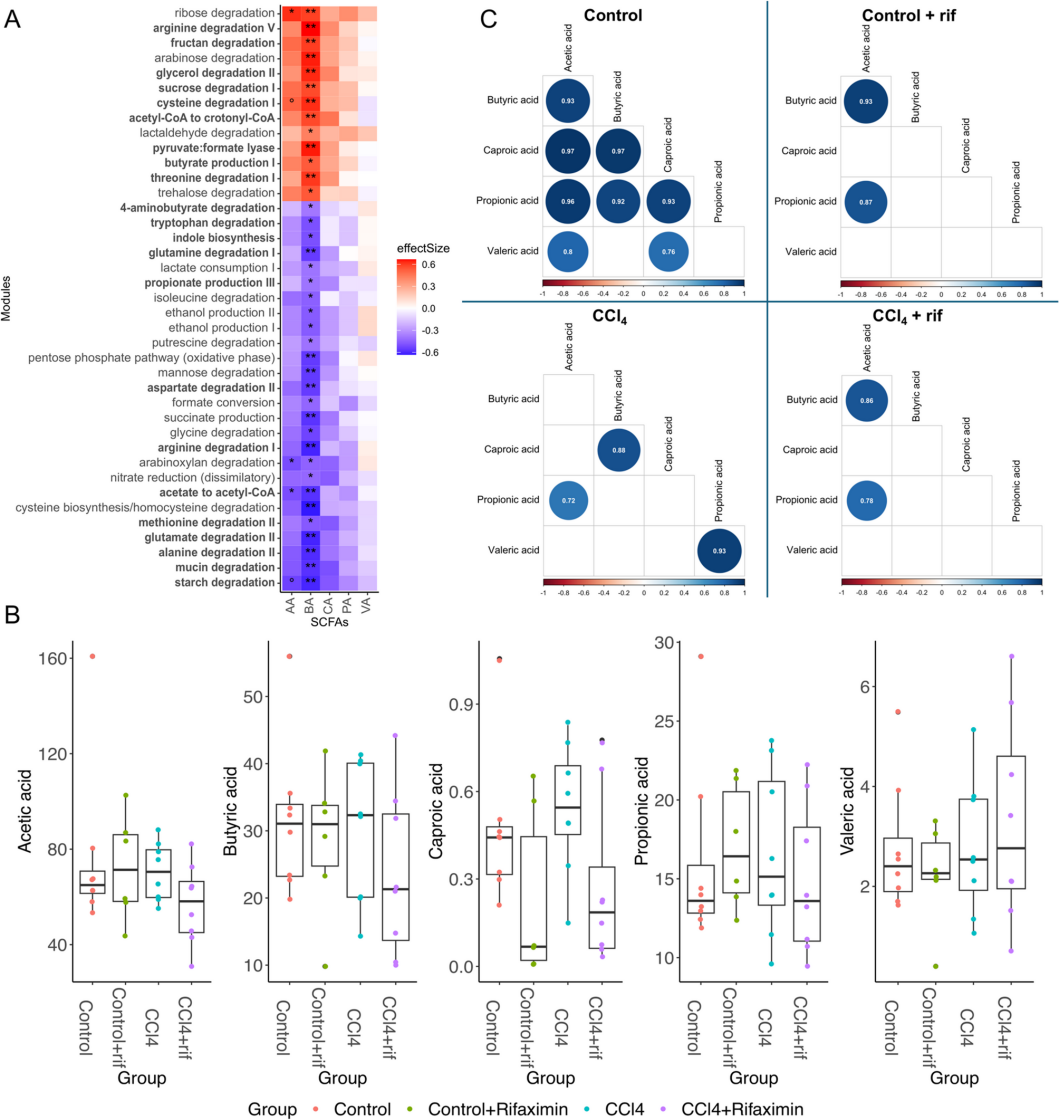
Relevance of SCFA metabolism. (A) Heatmap displaying the relationships between GMM (Gut Metabolic Modules) and short-chain fatty acids (SCFA), on the y and x-axis respectively. The color scale represents the magnitude of the effect size. The significance of each association is represented by black asterisks based on the FDR-adjusted p-values of the initial tests (FDR-values: < 0.001 = ***, < 0.01 = **, < 0.1 = *). AA, acetic acid; BA, butyric acid; CA, caproic acid, PA, propionic acid; VA, valeric acid. (B) Comparative analysis of SCFA between different groups (*n* = 8 per group). No asterisk is marked because of no statistical differences. (C) Correlation plots illustrating the relationship between SCFA in all the groups. Non-significant coefficients are depicted in blank, while significant correlations are shown by dots whose size and color depend on the correlation size.

To identify key predicted metabolic functions, significant correlations between metabolic modules identified by PICRUSt2 (KOs) and metadata variables were tested and classified under confounder correction with metadeconfoundR (see “Materials and methods” section for additional information). No metabolic changes could be associated with CCl_4_ treatment (FDR value > = 0.1). Only 2 significant modules were associated with antibiotic treatment: arabinoxylan degradation and methanol conversion (Supplementary Fig. 4). Not surprisingly, butyric acid was associated with the functional modules related to butyrate synthesis (including pyruvate formate lyase, acetyl-CoA to crotonyl-CoA), glycerol and saccharide utilization, and amino acid catabolism (arginine, cysteine and threonine) (Fig. 4A). Negative associations were found between butyric acid and several interesting gut metabolic modules (GMM) related to amino acid degradation (glutamate, glutamine, tryptophan, aspartate, methionine, alanine) and neurotransmitter or neuroactive-related modules such as indole biosynthesis, GABA degradation, mucin and starch degradation. Although not statistically significant, the associations observed between GMM and butyric acid were conserved with other SCFA (Fig. 4A).

The different groups of rats did not differ significantly in levels of each respective SCFA (Fig. 4B). In healthy rats without rifaximin intervention fecal levels of different SCFA within each sample remained significantly correlated even under overall variation, suggesting their relative levels are in homeostasis. In contrast, under liver injury and under rifaximin treatment, this correlation is lost as proportions between the different SCFA within each sample become dysregulated (Fig. 4C). The thus disrupted correlation pattern induced by rifaximin was similar in both healthy rats and rats with liver injury. This suggests that synthesis of the main SCFA is balanced in the gut microbiome under healthy conditions.

## Discussion

The use of the non-systemic antibiotic rifaximin, effectively reduces the risk of recurrent HE and restores cognitive performance in cirrhotic patients and in rats with induced-mild liver injury^6–8,11^. Our group previously found that administration of rifaximin prevents the infiltration of peripheral immune cells into the brain and the subsequent enhancement of neuroinflammation, changes in neurotransmission, and cognitive and motor impairment^5,6^. However, the mechanisms behind the rifaximin effect are largely unknown. To evaluate how this antibiotic primarily targets the gut, a selective pressure on the gut microbiota under liver disease, we used a rat model with induced liver injury and rifaximin treatment.

Antibiotic treatment is expected to affect microbial survival, resulting in a remarkable reduction in the α-diversity of the human gut microbiota^17,18^. Specifically, rifaximin inhibits bacterial RNA synthesis, and primarily targets clostridia and other enteropathogenic bacteria in murine models^19–22^. In this work, rifaximin treatment reduced α-diversity and 11 ASVs of SCFA producing bacteria in healthy rats, while in rats with induced liver injury, rifaximin induced complex changes affecting 68 ASVs belonging to the families *Bacteroidaceae*, *Ruminococcaceae* and *Lachnospiraceae*, and a significant reduction of *Clostridiaceae*, as previously described^19–21^. However, inflammation may play an additive role to rifaximin, leading to the inhibition of strong competitors, resulting in the growth of bacterial families that had a beneficial effect on the disease, but further studies are needed to understand the effects of rifaximin on the microbiome diversity.

To gain knowledge on which mechanisms might link the effect of gut microbiota on the cognition improvement mediated by rifaximin, we also explored taxa associations with neurobiological and metabolic measures. Looking at the general association patterns of the bacterial genera described here, it is evident that the column showing the antibiotic association pattern has high effect sizes and is very similar to the learning index and NR2A columns, while it is complementary or opposite to the association pattern of other variables, such as the membrane TNFα receptor, TNFR1. Membrane expression of glutamate receptor subunits and glutamate and GABA transporters, in particular the NMDA receptor subunit NR1 and the GABA transporter GAT1, showed a pattern similar to that of TNFR1, which was opposite to that found for the NR2A and NR2B receptor subunits. Thus, rifaximin treatment was the main confounding factor that strongly favoured certain bacterial groups and consequently the positive association between bacterial genera (e.g. *Lachnospiraceae A2*, *Erysipelotrichaceae* and *Dorea*) and neurotransmitter receptors and cognition. These parameters show a similar pattern of correlation with bacterial genera to that of the learning index, which may explain how rifaximin positively affects cognition, as NR2 subunits in the hippocampus are known to modulate spatial learning^23^. In fact, the family *Erysipelotricaceae* was found positively associated with cognitive function and *Lachnospiraceae* is the main predictor of fluid intelligence^24^ possibly because they metabolize glutamate and are efficient producers of SCFA.

SCFA are microbiota-derived metabolites influencing host physiology, especially in the gut-brain axis^14,15,25^. We analyzed fecal concentrations of butyrate, propionate, acetate, caproic and valerate. Interestingly, the concentrations of most of the SCFA analyzed were highly correlated in healthy rats, suggesting that the balance between the studied SCFA may be more important than their individual concentrations, which are discrepant between previous studies exploring liver disease and other conditions, such as Parkinson and obesity^26–30^. Despite the large volume of research devoted to the relationship between SCFA and intestinal health, the concept of an “SCFA balance” offers a novel perspective. This means that SCFA synthesis by the native gut microbiome maintains a balance associated with health, and liver disease and antibiotic intervention showed an altered SCFA balance. We hypothesize that maintaining a global balance of SCFA, which is managed by the gut microbiome, is an effective way to monitor intestinal health. Disruptions to this balance, such as those caused by liver disease or antibiotic intervention, contribute to intestinal dysbiosis and related health issues.

This work also aimed to investigate the changes on the gut microbiota functionality to infer the interaction with host metabolic functions. GMM represent cellular processes and are obtained using data from bacterial genome databases to prepare relational inferences to assess differential metabolic potential of bacteria. A negative correlation was found between butyrate levels and modules related to degradation of neuroactive related compounds, such as glutamine, tryptophan, alanine or glycine, and including two main neurotransmitters, GABA and glutamate. Impaired glutamate and GABA neurotransmission has been widely reported in rats with MHE, including rats with CCl_4_-induced liver injury^5,6,31^. Hyperammonemic rats with MHE also show an altered glycinergic system in the cerebellum. These results suggest that microbiota alterations, and especially bacterial production of SCFA in rats with liver injury and HE, can have a role in the induction of the described alterations of these neurotransmitter systems. Some reports described bacterial groups that modulate glutamate or GABA metabolism and associated these effects to neurological pathologies as Alzheimer disease or major depression^32,33^. Although GABA modulation by microbiota has been related to the GABA regulation of anxiety and depression, we suggest here that since GABA is also involved in cognitive function, these GABA modulating species must also affect cognitive function. However, the relevance of these pathways in terms of neurotransmitter availability needs to be confirmed in the context of liver disease and neurological impairment in general.

In conclusion, our study showed a different effect of rifaximin in healthy rats than in rats with induced liver injury, suggesting a possible combined effect of inflammation and rifaximin, inhibiting strong competitors and thus promoting the growth of bacterial families with a beneficial effect on the disease. Rifaximin appears to affect the interactions of the microbiota with alterations in neurotransmission, where *Dorea*,* Lachnospiraceae A2* and possibly *Erysipelotricaceae* would play a significant role. Changes in SCFA production by the microbiota in rats with liver injury may contribute to the alterations in neurotransmitter systems, including glutamate and GABA. Finally, we suggest a new concept, the “SCFA balance”, which represents a fundamental and unexplored aspect of gut microbiota research, critical for understanding gut microbiota dynamics in healthy and disease states. Our results contribute to a deeper understanding of the effects of rifaximin on the functional and taxonomic composition of the microbiome in liver injury.

## Materials and methods

### Experimental design

**Fig. 5:**
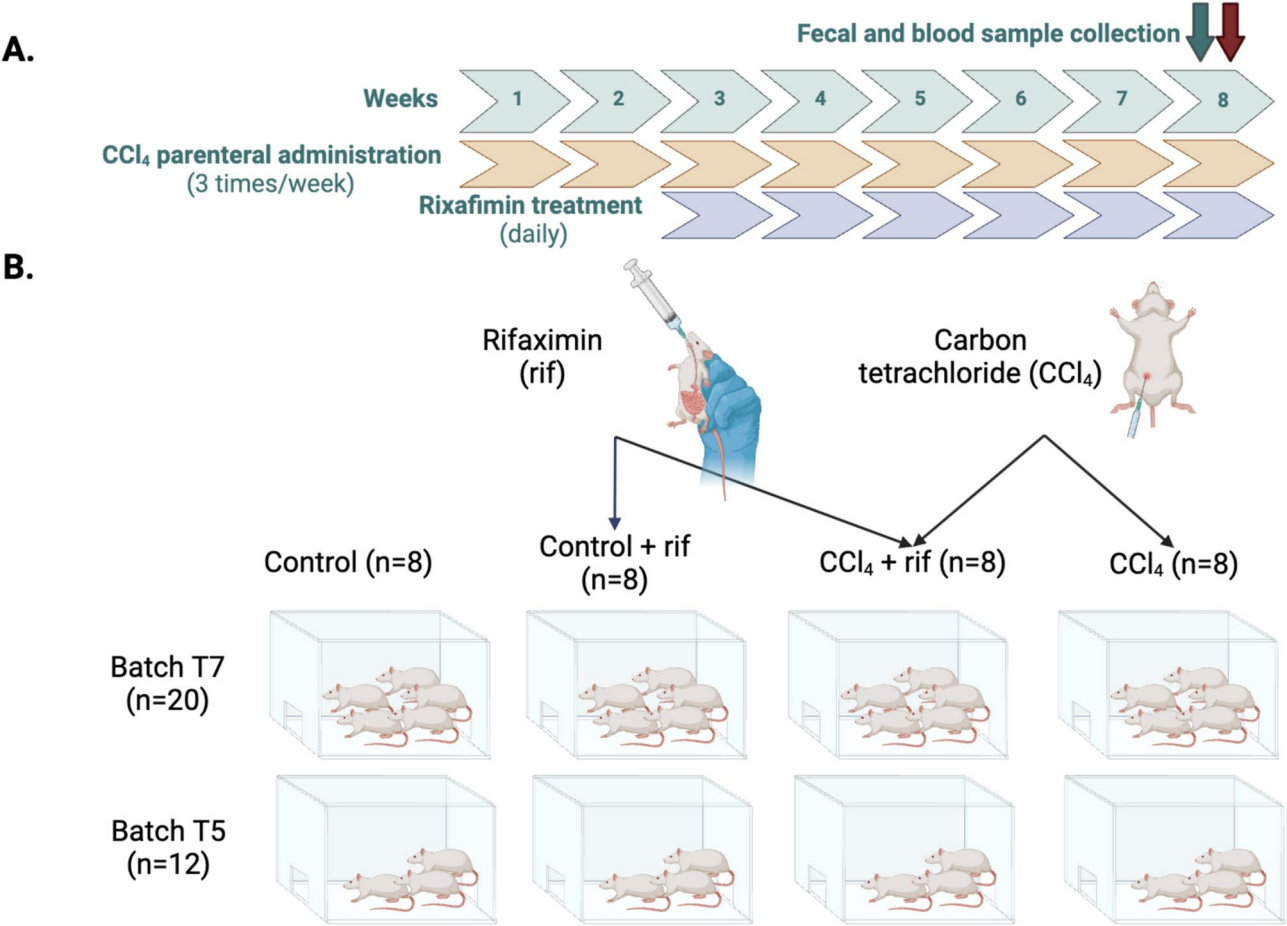
Diagram of experimental procedures. Fecal and blood samples were collected at the end of week 8. Treatment with rifaximin was only started after 2 weeks of CCl4 treatment (A). Schematic representation of the groups including the different working batches. Each group includes a total of eight rats, five from batch T7 and three from batch T5. (B). Created using BioRender.

Male Wistar rats (Charles River) weighing 150–180 g were intraperitoneally injected 3 times/week with 1 mL/kg body weight of CCl_4_ to induce liver injury. CCl_4_ was prepared 1:10 (v: v) in corn oil as in^4–6^. Control rats were intraperitoneally injected with corn oil. Rifaximin (Sigma, St. Louis, MO, USA) was dissolved in 100% ethanol and administered orally (20 mg/kg body weight). Two weeks after first CCl_4_ injection started daily rifaximin treatment and it was maintained until sacrifice after eight weeks of CCl_4_ administration. Control rats were orally treated with the same volume of 100% ethanol (Fig. 5A). Fecal samples were obtained at eight weeks, when rats weighed approximately 350 g. Four groups (*n* = 8 per group) were established: (1) control rats (2) control rats with rifaximin treatment, (3) rats with induced liver injury and (4) rats with induced liver injury treated with rifaximin. The experiment was run in two batches: T7 with 20 rats and T5 with 12 rats (Fig. 5B).

The experiments were approved by the Comité Ético de Experimentación Animal (CEEA) of Centro de Investigación Príncipe Felipe and the Conselleria de Agricultura de la Generalitat Valenciana (Valencian Government, Spain) and they were performed in accordance with the Directive of the European Commission (2010/63/EU) for the care and handling of experimental animals. All experiments were performed according the ARRIBE guideline.

### DNA extraction

Fecal samples were collected, flash-frozen in liquid nitrogen, and stored at -80 °C until analysis. Samples were resuspended in 100 µL PBS and homogenized by 30 s at low-ultrasound intensity bath in a sonicator VCI-50 (Raypa, Barcelona, Spain). DNA was extracted using the MagNA Pure LC DNA Isolation Kit III for Bacteria and Fungi (Roche Diagnostics) with a MagNA Pure LC 2.0 Instrument (Roche Diagnostics, Risch-Rotkreuz, Switzerland). The manufacturer’s instructions were followed, with an additional enzymatic lysis as in^34^. DNA was resuspended in 100 µL elution buffer and frozen at -20 °C until analysis. DNA concentration was quantified by fluorimetry, using the Quant-iT™ PicoGreen^®^ dsDNA Assay Kit in a Qubit™ 3 Fluorometer (both ThermoScientific).

### 16S rRNA gene bacterial profiling

An Illumina amplicon library was obtained according to the 16 S rRNA gene Metagenomic Sequencing Library Preparation Illumina protocol (Part #15044223 Rev. A). This targeted the 16 S rRNA gene V3 and V4 hypervariable regions, resulting in an amplicon of 460 bp. Amplicons were sequenced using the 2 × 300 bp paired-ends protocol on a MiSeq Sequencer (Illumina, San Diego, California, US), following the manufacturer’s instructions. Quality assessment and pre-processing of the sequences were performed following the DADA2 (v1.18.0)^35^ pipeline in R (v4.0.3) to infer amplicon sequence variants (ASVs) from the SILVA database (v138.1) with the following adjustments: primer sequences were trimmed from all reads. Forward and reverse reads were truncated to 290 and 265 bases, respectively. Fragments between 397 and 424 bp were selected (after trimming), based on the expected size of the 16 S amplicon. All other parameters were set to default. Taxonomic ASV abundance and sample data were compiled into a single object for further analysis using the phyloseq package (v1.38.0). The scripts used for the analysis can be found at https://github.com/lolapsgp/Rats_HE_CCl4model.git. All statistical analyses were conducted using R (v4.1.2).

Low-prevalence ASVs that were not present in at least 50% of the samples in any of the groups were removed, with each group consisting of eight rats. The resulting requirement is that selected ASVs must have four non-zero values in at least one group (Supplementary Tables 1, 2 and 3). Finally, the data were compositionally transformed (relative abundances).

### Alpha- and beta-diversity

Alpha-diversity indices (Shannon, Chao1, and Fisher) were calculated using raw sample counts since all samples reached saturation when performing rarefaction curve analysis. To assess whether there were statistical differences between the different groups and to further take into account batch-related effects, a linear mixed-effects modeling approach was adopted. Two models were compared: the first included only the batch effect, (*Diversity index ~ (1|Batch))*, while the second model incorporated both the batch effect and group information (*Diversity index ~ Group + (1|Batch))*. This comparison aimed to determine whether the group variable significantly contributed to the model’s fit. The data was further subdivided into groups and analyzed for specific group comparisons. To address the problem of multiple comparisons and to control for false discovery rates, the p-values of the results obtained were calculated using the p.adjust function and adjusted with the Benjamini-Hochberg (BH) method.

Filtered and compositionally transformed taxa were classified as dominant by a cutoff higher than 0.1 regarding relative abundance. All plots were obtained using ggplot2 package (v3.4.2). The filtered data were transformed using logarithmic conversion and ARSyN function^36^ from the MultiBaC^37^ package in R (v4.1.2)^38^ to remove the batch effect for β-diversity analysis. The parameters were set as default. ARSyN functions as a Batch Effect Correction Algorithm (BECA). It uses an analysis of variance (ANOVA) decomposition to the data matrix for estimating the batch effect and, subsequently, a Principal Component Analysis (PCA) is applied to each submatrix to estimate the systematic batch-induced variation, which is then removed from the original data. For the visualization of β-diversity, the vegdist function of the vegan package (v2.6.4) was used to compute Bray-Curtis distances, to which Principal Coordinates Analysis (PCoA) ordination was then applied and used for plotting. PERMANOVA was performed for multivariant comparisons using adonis function of the vegan package (v2.6.4), specifically, tests were performed by marginal effects, 999 permutations were defined, and stratification by batch was applied. Diversity analyses were carried out to find out the main causes of modifying the composition of the microbiome.

### Predicted functional annotation

PICRUSt2 algorithm (Phylogenetic Investigation of Communities by Reconstruction of Unobserved States 2) (https://github.com/picrust/picrust2)^39^ was used to predict the functional potential based on the taxonomic information. Briefly, PICRUSt2 maps the ASVs into a phylogenetic tree based on 20,000 available bacterial whole genomes and then estimates the abundance of predicted metagenomes, KEGG Orthologs (KO)^40,41^ and pathways. KO counts were binned to GMM, which are sets of alternative KO combinations representing a cellular process. This was done using omixer-rpmR, which is an R interface to Omixer-RPM a tool that selects modules that pass a defined coverage cutoff (set by default) and chooses the combination of KOs that maximizes the abundance of the module^42^. For better coverage of the tryptophan (trp) metabolism, the following modules were manually added to the Omixer-rpmR bundled databases based on KEGG modules and by Kaur et al.^43^ for pathway prediction criteria: kynurenine biosynthesis I (K00453, K00463, K01432, K14263, K07130, K00486, K01556, K00452, K03392, K10217, K23234); melatonin biosynthesis I (K00502, K01593, K00669, K00543); quinolinic acid biosynthesis I (K00453, K00463, K01432, K14263, K07130, K00486, K01556, K00452, K00767, K00969, K06210, K01916, K01950); indole biosynthesis (K01667); indole propionic acid from trp (K13607); tryptamine propionic acid from trp (K01593).

### Differential abundance analysis

The R package MetadeconfoundR^44^ version 0.2.8 was used to distinguish changes related to the disease or the treatment, specifically, for differential abundance analysis and covariate deconfounding on each metagenomics feature (taxa and functional modules). MetadeconfoundR uses mixed-effects linear models to infer significant associations between microbiome features and the variables in the metadata (FDR < 0.1). All significant associations were corrected for any potential confounding variables (third variable related to a given microbiome feature). The batch variable was included as a random variable. Comparisons were performed at ASV level and genus level, involving two main groups: rats treated with rifaximin versus rats not receiving antibiotic treatment. In addition, data were segregated for specific group comparisons, contrasting the effect of antibiotic treatment in healthy and liver-damaged rats separately. To assess and visualize the correlations within metadata variables, ggpairs and ggcorr functions were employed (package GGally v2.1.2) and ggplot function from package ggplot2 package (v3.4.2).

### Analysis of SCFA in feces

The concentration of SCFA in feces was measured by LC-MS with an EXION (Shimatzu) HPLC system coupled to a mass spectrometry detection system consisting of a QTRAP 4500 triple quadrupole (AB Sciex, Ontario, Canada) equipped with electrospray ionization (ESI) ion source, controlled by the Analyst software, version 1.6.3. The sample preparation proceeded as follows: around 100 µg of feces of each rat were homogenized in 2 mL of LC-MS grade water (1:20 p/v) for three minutes in a Polytron-Aggregate PT 1200 E (Kinematica, Luzern, Switzerland) and centrifuged at 10000*g* for 10 min at 4 °C. Sample derivatization and extraction method was modified from^45^ as follows: 30 µL of supernatant were derivatized with 10 µL of 1 M O-benzylhydroxylamine (O-BHA) (SIGMA) and 10 µL of 1 M N-(3-Dimethylaminopropyl)-N-ethylcarbodiimide-HCl (EDC) (SIGMA) prepared in freshly prepared pyridine buffer (270 µL of 12.1 M HCl + 430 µL of pyridine in H2O to 5 mL, pH 5), in a fume hood. This derivatization mixture was agitated at 300 rpm for 10 min. Thereafter 450 µL of 50% methanol in water were added to dilute samples 1:10. Samples were vortexed and 300 µL of dichloromethane were added to 100 µL of derivatized sample for extraction by agitation at 300 rpm for 30 min. Then, 100 µL of the organic phase was separated and evaporated at room temperature in a fume hood. The samples were reconstituted with 85 µL of 0.1% formic acid in H2O and 40 µL and injected in the HPLC under the following conditions: a Kinetex C18 100*4.6 mm 2.6 column from Phenomenex, at 40º C, was used. The mobile phase consisted of a two-phase gradient: 0.1% formic acid in water (A) and 0.1% formic acid in acetonitrile (B), as follows: 5–20% B 0–1 min, 20–50% B 1–5.5 min, 60% B 5.5–5.7 min, 80% B 6 min, 80% B 6.5 min, 5% B 6.6 min, 5% B 12 min, with a flow rate of 0.4 mL/min. The conditions of the mass spectrometer were: positive ionization mode, entrance potential 10, curtain gas 30, declustering potential 60 V, collision energy 15 eV, GAS1 40 and GAS2 50, 500 °C and 4500 V in multiple reaction monitoring (MRM) mode with the following transitions for the quantification of the different SCFA: acetic acid 166.1 m/z > 91 m/z; propionic acid 180.1 m/z > 91 m/z; butyric acid and isobutyric acid 194.1 m/z > 91 m/z; valeric acid 208 m/z > 91 m/z and caproic acid 222 m/z > 91 m/z. A SCFA mixture standard curve, from 1 to 2500 ng/mL (10 to 25000 ng/mL of caproic acid), was prepared in H2O and derivatized and extracted as the samples to calculate SCFA concentrations.

## Supplementary Information

Below is the link to the electronic supplementary material.

Supplementary Material 1

Supplementary Material 2

Supplementary Material 3

Supplementary Material 4

Supplementary Material 5

Supplementary Material 6

Supplementary Material 7

Supplementary Material 8

Supplementary Material 9

Supplementary Material 10

Supplementary Material 11

Supplementary Material 12

## Data Availability

The scripts used for the analysis can be found at https://github.com/lolapsgp/Rats_HE_CCl4model.git. The sequencing data that support the findings of this study are available as a SRA at NCBI with submission code SUB14548047: https://www.ncbi.nlm.nih.gov/bioproject/PRJNA1125883. All other data are included in the study in the Supplementary Data section as Supplementary Files.
