# Supplementary figures and images for: Rifaximin-induced changes in the gut microbiome associated to improvement of neurotransmission alterations and learning in rats with chronic liver disease
Source: Sci Rep. 2025 Oct 2;15:34382. doi: 10.1038/s41598-025-17229-1 (PMC12491630; doi:10.1038/s41598-025-17229-1)

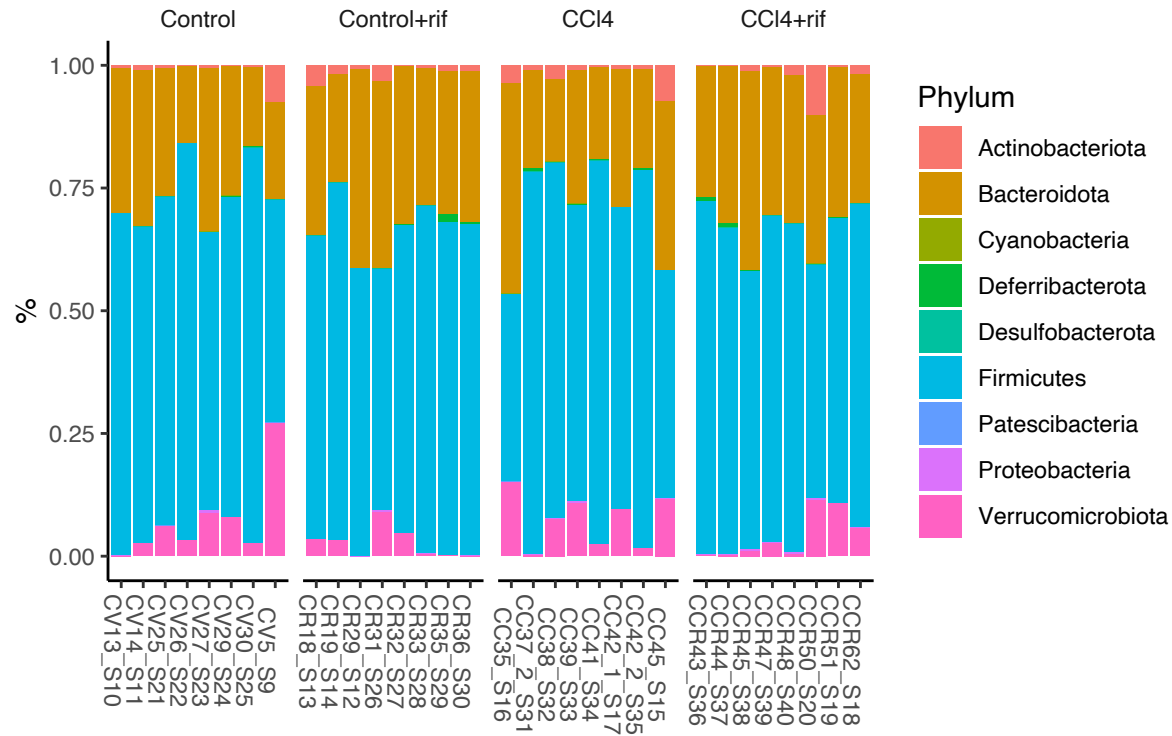

Supplement: Supplementary file 1 — Supplementary Material 1 [file 41598_2025_17229_MOESM1_ESM.pdf]

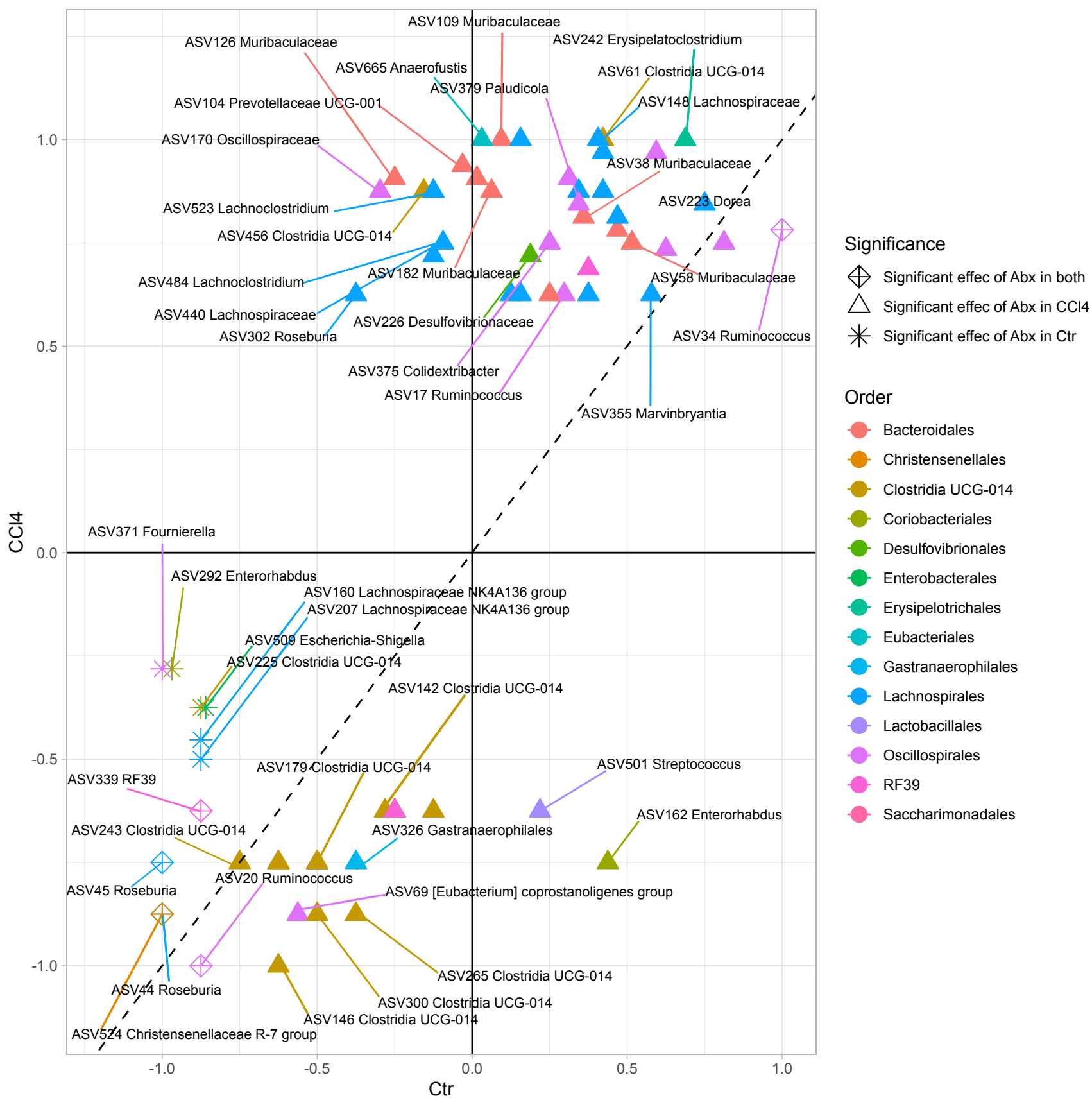

Supplement: Supplementary file 2 — Supplementary Material 2 [file 41598_2025_17229_MOESM2_ESM.pdf]

Significant metadata correlation heatmap

FDR-values: < 0.001 = \*\*\*, < 0.01 = \*\*, < 0.1 = \*

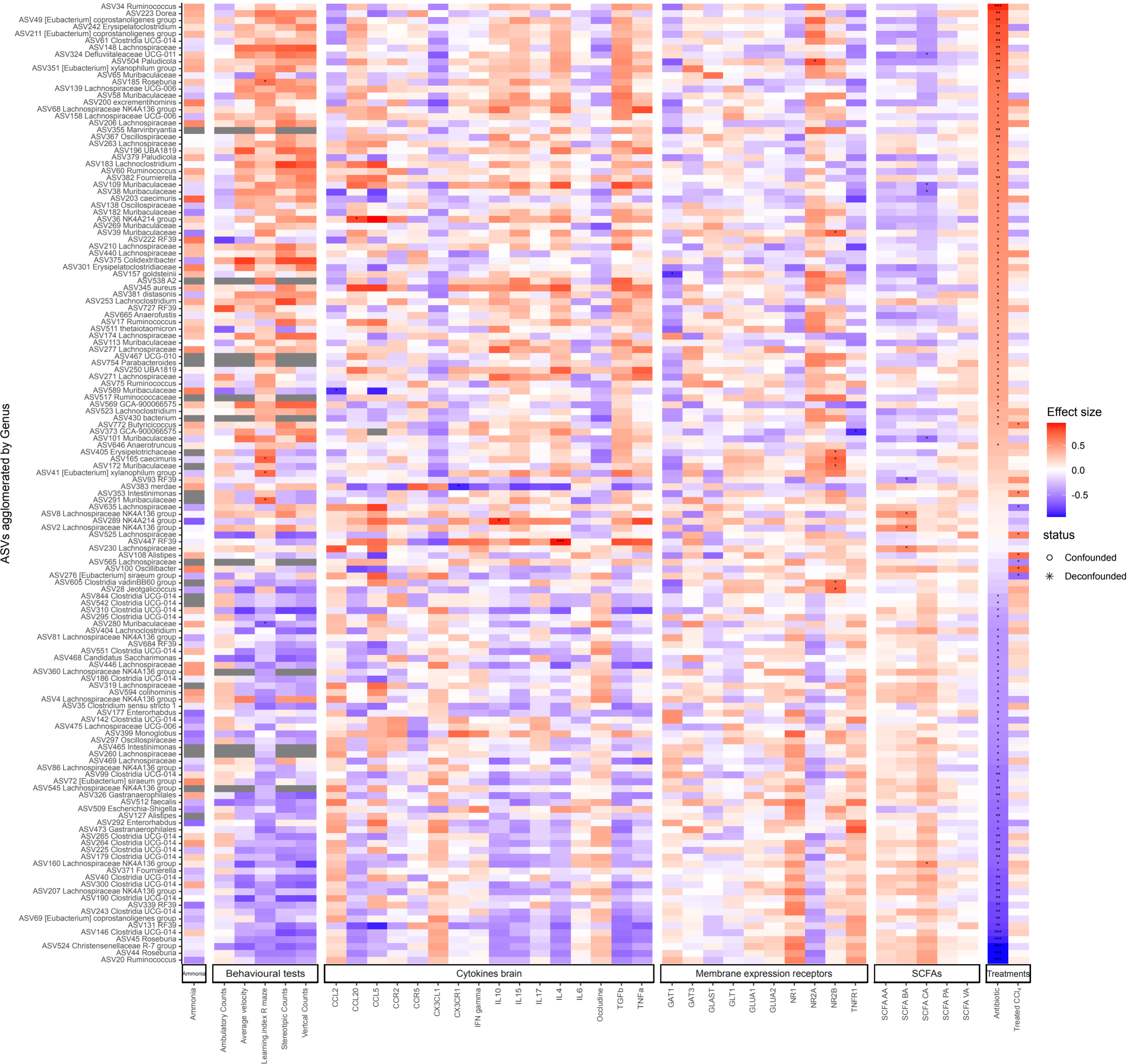

Supplement: Supplementary file 3 — Supplementary Material 3 [file 41598_2025_17229_MOESM3_ESM.pdf]

Summarizing heatmap

FDR-values: < 0.001 = \*\*\*, < 0.01 = \*\*, < 0.1 = \*

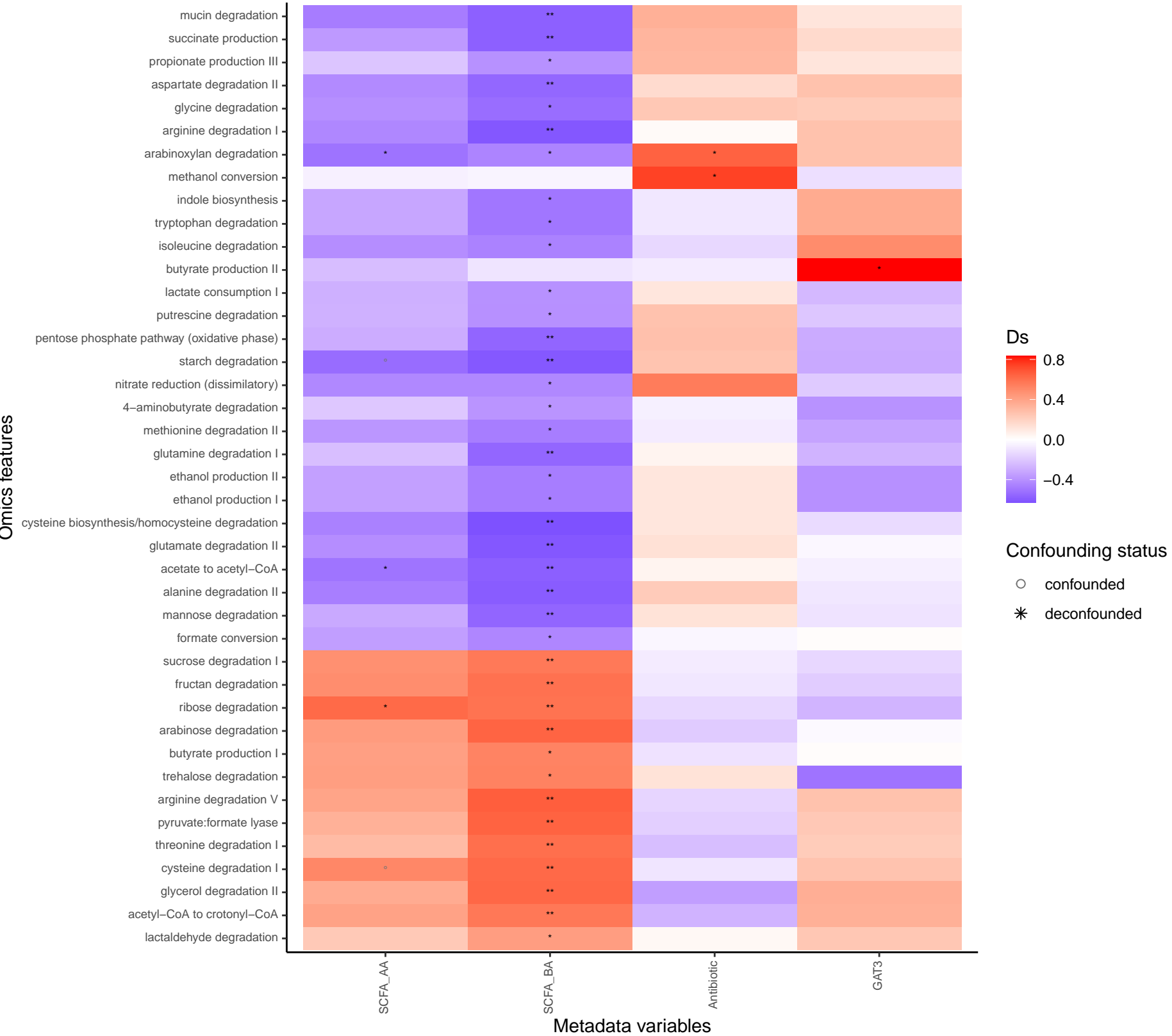

Supplement: Supplementary file 4 — Supplementary Material 4 [file 41598_2025_17229_MOESM4_ESM.pdf]
