# Supplementary material for: Rifaximin-induced changes in the gut microbiome associated to improvement of neurotransmission alterations and learning in rats with chronic liver disease
Source: Sci Rep. 2025 Oct 2;15:34382. doi: 10.1038/s41598-025-17229-1 (PMC12491630; doi:10.1038/s41598-025-17229-1)
Supplement: Supplementary file 10 — Supplementary Material 10 [file 41598_2025_17229_MOESM10_ESM.docx]

| Bacteria | Antibiotic | Butyric acid | TNFR1 | NR1 | NR2A | CCL20 | Learning index (Rmaze) |
| --- | --- | --- | --- | --- | --- | --- | --- |
| Gastranaerophilales | - (conf) |  | + |  |  |  |  |
| Clostridia UCG-014 | - (conf) |  |  |  | - |  |  |
| Oscilospiraceae NK4A214 | + (conf) |  |  |  |  | + |  |
| *Monoglobus* | - |  |  | + (conf) |  |  |  |
| *Marivinbryantia* | + (conf) |  |  |  | + |  |  |
| *Frisingiococcus* | + |  | - (conf) |  |  |  |  |
| Erysipelotrichaceae | + |  |  |  | + |  | +  (conf) |
| Erysipelatoclostridiaceae | + | - |  | -  (conf) |  |  |  |
| *Enterococcus* | - (conf) |  |  | + |  |  |  |
| *Clostridium sensu stricto 1* | - (conf) |  |  |  |  |  | - |

**Supplementary table 4. Confounded associations between bacterial genera and metavariables.** This table highlights those confounded associations identified using metadeconfoundR by other variables. The red color shows a positive relationship between the sign of correlations to the bacteria and the blue color a negative relationship. Short-chain fatty acids (SCFAs) are excluded from this table due to their expected mutual associations.
