# Supplementary material for: Rifaximin-induced changes in the gut microbiome associated to improvement of neurotransmission alterations and learning in rats with chronic liver disease
Source: Sci Rep. 2025 Oct 2;15:34382. doi: 10.1038/s41598-025-17229-1 (PMC12491630; doi:10.1038/s41598-025-17229-1)
Supplement: Supplementary file 11 — Supplementary Material 11 [file 41598_2025_17229_MOESM11_ESM.docx]

**Rifaximin-induced changes in the gut microbiome associated to improvement of neurotransmission alterations and learning in rats with chronic liver disease**

Lola Giner-Pérez^1,2^, Víctor Hugo Jarquín-Díaz^3,4,5^, Paola Leone^2*^, Carla Giménez-Garzó^2*^, Gergana Mincheva^2^, Álex Mira^6^, Sofia Kirke Forslund-Startceva^3,4,5^, Teresa Rubio^2^, Vicente Felipo^2^, Gaspar Pérez-Martínez^1^ and Marta Llansola^2#^

**Supplementary methods**

***Cytokine content by western blot***

Animals were euthanized by decapitation after 8 weeks of CCl_~~4~~_ administration and six weeks of daily rifaximin treatment. Hippocampus was dissected and homogenized in five volumes of lysis buffer (50 mM TRIS–HCl pH 7.5, 50 mM NaCl, 10 mM EGTA, 5 mM EDTA and protease and phosphatase inhibitors) by sonication and after centrifugation at 13000xg for 10 min total protein content was determined in the supernatant by the bicinchoninic acid method (BCA), from Pierce (Rockford, IL, USA). Samples were subjected to electrophoresis and immunoblotting using the following primary antibodies: TNFα (AF-510-NA) 1: 500 and IFN-gamma (MAB5851) 1:1000 from R&D SYSTEMS( Minneapolis, MN, USA), IL-17 (ab79056), IL-4 (ab9811), IL-10 (ab9969), CCl20 (ab9829), CCR5 (ab65850) and CX3CR1 (ab8021), all 1:1000, from Abcam (Cambridge, UK); TGFβ (PA5-99186) 1:1000, CX3CL1 (14-7986) 1:1000, and CCL5 (710001) 1:500 from Invitrogen (Waltham, MA, USA); IL-6 (ARC0062) 1:500 from BioSource (Camarillo, CA, USA); IL-15 (1829R) 1:200 from BIOSS (Woburn, MA, USA); CCL2 (66272) 1:1000 from Proteintech (Manchester, UK) and CCR2 (NBP1-48337) 1:1000 and Occludine (NBP1-87402) 1:2000 from Novus Biologicals (Centennial, CO, USA). GAPDH (1:15000, MAB374, Millipore (Burlington, MA, USA) or β-actin (1:5000, ab6276, Abcam) were used as a loading control. Secondary antibodies were anti-rabbit, anti-goat, or anti-mouse IgG, 1:4000 dilution (cat. #A8025, A7650, and A3562, respectively), conjugated with alkaline phosphatase from Sigma (St. Louis, MO, USA). The images were captured using ScanJet 5300C (Hewlett-Packard, Amsterdam, the Netherlands) and the band intensities quantified using ΑlphaImager 2200, version 3.1.2 (Αlpha Innotech, Watertown, MA, USA).

***Membrane expression***

It was analyzed as described in [1]. Rats were euthanized by decapitation and each dissected hippocampus was put into ice-cold Krebs buffer (in mmol/L): NaCl 119, KCl 2.5, KH2PO4 1, NaHCO3 26.2, CaCl2 2.5, and glucose 11, aerated with 95% O2 and 5% CO2 at pH 7.4. Transversal 400 µm thick slices were obtained with a chopper. Slices were added to tubes containing ice-cold Krebs buffer with or without 2 mM bis(sulfosuccinimidyl)suberate (BS3) (Pierce, Rockford, IL, USA) and incubated for 30 min at 4 ºC. Cross-linking was terminated by adding 100 mM glycine (10 min, 4ºC). The slices were homogenized by sonication for 20 s. Samples treated with or without BS3 were analyzed by Western blot using antibodies against NR1 (NMDA Receptor subunit 1) (BD 556308) 1:1000 from BD Biosciences (Franklin Lakes, NJ, USA), NR2B (NMDA Receptor subunit 2B) (06-600) 1:1000, NR2A (NMDA Receptor subunit 2A) (04-901) 1:1000, GluA1 subunit of AMPA receptor (04-855) 1:1000 and GluA2 subunit of AMPA receptor (AB1768-I) 1:2000 all from Millipore, glutamate transporter 1 (GLT1) (PA5-19706) 1:1000 from Invitrogen, glutamate transporter GLAST (NB100-1869) 1:4000 from Novus Biologicals, GABA transporters GAT1 (ab426) 1:500 and GAT3 (ab431) 1:500 and TNFR1 (ab19139) 1:1000 from Abcam. The surface expression of receptor subunits was calculated as the difference between the intensity of the bands without BS3 (total protein) and with BS3 (non-membrane protein).

***Motor activity***

For evaluation of spontaneous motor activity an Actimeter (Med Associates) was used. The animals were placed in an open-field activity chamber (43 × 43 × 30.5 cm) and allowed to explore for 60 min. Activity was detected by arrays of infrared motion detection, with two arrays 1 cm above the floor of the chamber and another array 6 cm above the floor. The chambers were controlled by the Activity Monitor software (Med Associates), which record motor activity every 5 min. The software record data of different activity parameters including those we analysed: ambulatory, vertical (rearing), velocity and stereotypic movements.

***Determination of ammonia in blood***

Blood was collected from the saphenous vein. Twenty microliters of blood were immediately used to measure ammonia with the Ammonia Test Kit II for the PocketChemBA system (Arkay, Inc., Kyoto, Japan).

1. Cabrera-Pastor, A. et al. In vivo administration of extracellular cGMP normalizes TNF-α and membrane expression of AMPA receptors in hippocampus and spatial reference memory but not IL-1β, NMDA receptors in membrane and working memory in hyperammonemic rats. Brain Behav Immun 57, 360–370 (2016).

**Supplementary Figure legends**

**Supplementary Figure 1.** Barplots showing the relative abundance of microbial phyla in different samples. Each bar represents one sample and shows the distribution of phyla within the microbiome.

**Supplementary Figure 2.** Venn Diagram displaying ASVs associated with rifaximin treatment by group. This figure displays the significant relationships between ASVs and rifaximin treatment in healthy rats and rats with mild liver damage (CCl4), tested separately (n=8 per group). The colors represent if the correlation between the ASV and rifaximin was positive (red) or negative (blue).

**Supplementary Figure 3.** Heatmap displaying significant relationships between ASVs (Amplicon Sequence Variants) and all the meta-variables, on the y and x-axis respectively. The colour scale on the heatmap represents the magnitude of the effect size. Significance is denoted by black asterisks based on FDR-adjusted p-values, with gray circles representing associations that, although significant, are confounded. SCFA, short chain fatty acid; AA, acetic acid; BA, butyric acid; CA, caproic acid; PA, propionic acid; VA, valeric acid.

**Supplementary Figure 4.** Heatmap displaying the significant relationships between GMMs and metavariables, on the y and x-axis respectively. The color scale represents the magnitude of the effect size. The significance of each association is represented by black asterisks based on the FDR-adjusted p-values of the initial tests. SCFA, short chain fatty acid; AA, acetic acid; BA, butyric acid.

**Legends for Supplementary Files and Tables**

**Supplementary File 1.** Metadata information for all the samples.

**Supplementary File 2.** MetadeconfoundR results. Raw p-values (sheet “Ps”), multiple testing corrected p-values (sheet “Qs”), corresponding effect size (sheet “Ds”), and confounding status (sheet “status”) of the correlations between the variables and the features (ASVs). The data is subsetted by rats with mild liver damage (CCl_4_) and CCl_4_ rats treated with rifaximin.

**Supplementary File 3.** MetadeconfoundR results. Raw p-values (sheet “Ps”), multiple testing corrected p-values (sheet “Qs”), corresponding effect size (sheet “Ds”), and confounding status (sheet “status”) of the correlations between the variables and the features (ASVs). The data is subsetted by control rats and control rats treated with rifaximin.

**Supplementary Table 1.** Number of identifies ASVs before and after filtering and final number of reads kept by group.

**Supplementary Table 2.** Phyla present in the data ordered from most abundant to lowest (dominant), calculated with dominant function of the microbiome package.

**Supplementary Table 3.** Families present in the data ordered from most abundant to lowest (dominant), calculated with dominant function of the microbiome package.

**Supplementary Table 4.** Confounded associations between bacterial genera and metavariables. This table highlights those confounded associations identified using metadeconfoundR by other variables. The red colour shows a positive relationship between the sign of correlations to the bacteria and the blue colour a negative relationship. Short-chain fatty acids (SCFAs) are excluded from this table due to their expected mutual associations.
